# Age-Related Modulations of AQP4 and Caveolin-1 in the Hippocampus Predispose the Toxic Effect of *Phoneutria nigriventer* Spider Venom

**DOI:** 10.3390/ijms17111462

**Published:** 2016-11-23

**Authors:** Edilene S. Soares, Leila M. Stávale, Monique C. P. Mendonça, Andressa Coope, Maria Alice da Cruz-Höfling

**Affiliations:** 1 Department of Biochemistry and Tissue Biology, Institute of Biology, State University of Campinas, Campinas, SP 13083-863, Brazil; esiqueirasoares@gmail.com (E.S.S.); leilinhabio@yahoo.com.br (L.M.S.); mo_padilha@hotmail.com (M.C.P.M.); 2 Department of Pharmacology, Faculty of Medical Sciences, State University of Campinas, Campinas, SP 13083-887, Brazil; 3 Laboratory of Cell Signaling, Faculty of Medical Sciences, State University of Campinas, Campinas, SP 13083-887, Brazil; andressacoope@gmail.com

**Keywords:** caveolin-1, aquaporins, hippocampus, spider venom

## Abstract

We have previously demonstrated that *Phoneutria nigriventer* venom (PNV) causes blood–brain barrier (BBB) breakdown, swelling of astrocytes end-feet and fluid permeation into brain interstitium in rats. Caveolae and water channels respond to BBB alterations by co-participation in shear stress response and edema formation/resolution. Herein, we showed post-natal developmental-related changes of two BBB-associated transporter proteins: the endothelial caveolin-1 (Cav-1), the major scaffolding protein from caveolae frame, and the astroglial aquaporin-4 (AQP4), the main water channel protein expressed in astrocytic peri-vascular end-feet processes, in the hippocampus of rats intraperitoneally-administered PNV. Western blotting protein levels; immunohistochemistry (IHC) protein distribution in CA1, CA2, and CA3 subfields; and gene expression by Real Time-Polymerase Chain Reaction (qPCR) were assessed in post-natal Day 14 (P14) and 8–10-week-old rats over critical periods of envenomation. The intensity and duration of the toxic manifestations indicate P14 neonate rats more vulnerable to PNV than adults. Histologically, the capillaries of P14 and 8–10-week-old rats treated with PNV showed perivascular edema, while controls did not. The intensity of the toxic manifestations in P14 decreases temporally (2 > 5 > 24 h), while inversely the expression of AQP4 and Cav-1 peaked at 24 h when clinically PNV-treated animals do not differ from saline controls. IHC of AQP4 revealed that hippocampal CA1 showed the least expression at 2 h when toxic manifestation was maximal. Subfield IHC quantification revealed that in P14 rats Cav-1 peaked at 24 h when toxic manifestations were absent, whereas in 8–10-week-old rats Cav-1 peaked at 2 h when toxic signs were highest, and progressively attenuated such increases until 24 h, remaining though significantly above baseline. Considering astrocyte-endothelial physical and functional interactions, we hypothesize that age-related modulations of AQP4 and Cav-1 might be linked both to changes in functional properties of astrocytes during post-natal development and in the BBB breakdown induced by the venom of *P. nigriventer*.

## 1. Introduction

Accidents involving the spider *Phoneutria nigriventer* are common in the Southeast of Brazil. They are responsible for great part of the notified cases of araneism in the country; the majority of accidents being characterized by intense local pain and edema which rarely evolves to serious complications or death. Less than 1% of accidents are graded as severe, children being the main victims of this category of accident [1]. The *P. nigriventer* venom (PNV) produces important excitatory effects [2,3] and its mechanism of action indicates the nervous system a key target. A plethora of neurotoxic peptides contained in the venom shows activity on Na^+^, K^+^ and Ca^2+^ channels and chemical receptors of excitable membranes of insects and mammals ([4,5], see [6] for review). It is known that altered homeostasis in ion movements in the nervous tissue compartments underlies neuronal dysfunction and a number of pathologies of the nervous system. Thereupon, a great therapeutic interest motivates the abundant number of physiological and pharmacological studies involving PNV aiming at the development of new drugs [6,7].

An important feature of PNV that has been consistently detected since the very first work by Le Sueur et al. [8] is its ability to disrupt the blood–brain barrier (BBB) in the hippocampus of rats. Apart from breaking down the paracellular barrier [7], PNV also disturbs the steady-state of the transcellular route by a microtubule-mediated transendothelial vesicular transport [9]. In addition to BBB disruption, PNV induces cytotoxic edema of perivascular astrocytes end-feet and vasogenic edema within neuropil interstitium in hippocampus [8] and cerebellum [10,11].

More recently, two proteins involved in transport mechanisms were shown to be upregulated in the cerebellum of animals administered with PNV, aquaporin-4 (AQP4) and caveolin-1 (Cav-1) [12,13,14]. AQP4 is one of the key players in edema formation and resolution [15]; it belongs to a family of integral transmembrane water channel proteins, which regulates the rapid transport of water within the perivascular astrocyte end-feet [16,17] thus permitting water selective bidirectional movement in response to osmotic gradient, without expenditure of energy (ATP) [18,19]. Cav-1 is the main structural protein of caveolae, dynamic cell structures that play role in endocytosis and trafficking via fluid-phase transcytosis [20]; Caveolae/caveolin-1 system also participates in lipid homeostasis, cellular/synaptic signaling [21,22,23,24]. Cav-1 is the most known protein of the caveolin family, being its α isoform more abundant in brain vessels [25]. In the brain, the increases in Cav-1 and endothelial caveolae induced by PNV [13,14] have been associated with BBB and pathologies of brain edema involving aquaporins [26,27].

The present study has been designed to investigate whether PNV affects two specific markers of key cells that form the BBB: the AQP4 of astrocytes and Cav-1 of endothelium. The investigation was carried out in the hippocampus of rats at different postnatal developmental stages, postnatal day fourteenth rats (P14) and 8–10 week-old rats. Time-course changes in AQP4 and Cav-1 expressions in the hippocampus were selected according to the clinical evolution of animals after envenomation: severe envenomation state (2 h), signs of amelioration of the clinical condition (5 h), and absence of signs of envenomation (24 h).

## 2. Results

The chronology of the clinical signs and symptoms of animals resulting from PNV injection followed the already described manifestations [8,10]. Soon after envenomation, the animals exhibited severe toxicity which was characterized by piloerection, hyper salivation, intense redness of the ears, paralyses of hind limbs, respiratory distress and stayed motionless at the cage corner; 5 h after PNV exposure, the animals started showing some signs of amelioration of their toxic condition; and 24 h after envenomation, the animals behavior were not different from the exhibited by the saline-treated animals. The severity of symptoms was more precocious and recovery delayed in P14 than in adult rats.

### 2.1. Western Blotting (WB) and Real Time-Polymerase Chain Reaction (qPCR)

**Figure 1:**
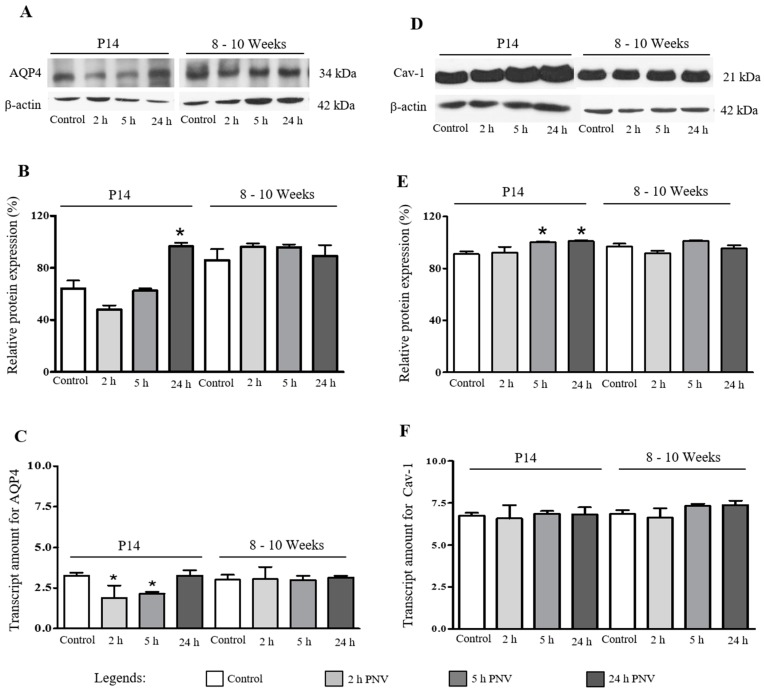
Expression of aquaporin (AQP4) and caveolin-1 (Cav-1) in hippocampus of 14-day-old and 8–10 weeks-old rats after 2, 5, and 24 h of 0.9% saline (control) or *Phoneutria nigriventer* venom (PNV) (1.7 mg/kg) i.p injection. (**A**,**D**) Representative Western blots for AQP4, Cav-1, and β-actin expression (endogenous control); (**B**,**E**) Graphic of Western Blotting for AQP4 and Cav-1. The results were shown as percentage of control (100%); (**C**,**F**) AQP4 and Cav-1 mRNA levels measured by qPCR were normalized by using GADPH as endogenous control. * *p* < 0.05 compared to the respective control (Student’s *t*-test of data expressed as mean ± SEM); *n* = 5/time point.

These assays were employed to determine the levels of AQP4 and Cav-1 proteins and respective mRNA transcripts in hippocampi of saline- and PNV-administered rats (Figure 1). In P14 neonate PNV-treated group, the expression of AQP4 showed a tendency to decrease followed by significant increase above baseline at 24 h (Figure 1A,B) (*p* < 0.05); AQP4 mRNA decreased at 2 and 5 h followed by recovery to baseline at 24 h (Figure 1C). Adult animals showed no alterations in both proteins and AQP4 mRNA (Figure 1A–C).

In regard to Cav-1, WB assay showed slight but significant up-regulation at 5 and 24 h post-PNV administration in neonate group (*p* < 0.05) while no differences were found between control and PNV-treated groups in adult rats (Figure 1D,E). Likewise, no differences were observed in mRNA levels in both ages (Figure 1F). The alterations in AQP4 and Cav-1 in neonate rats were significantly more prominent than in adult rats.

### 2.2. Immunohistochemistry (IHC)

IHC assays were used to determine possible variability in the level of AQP4 and Cav-1 per sub-field of hippocampus. IHC adds new information about differential regional alterations in addition to provide information about location and cellular distribution of the proteins.

**Figure 2:**
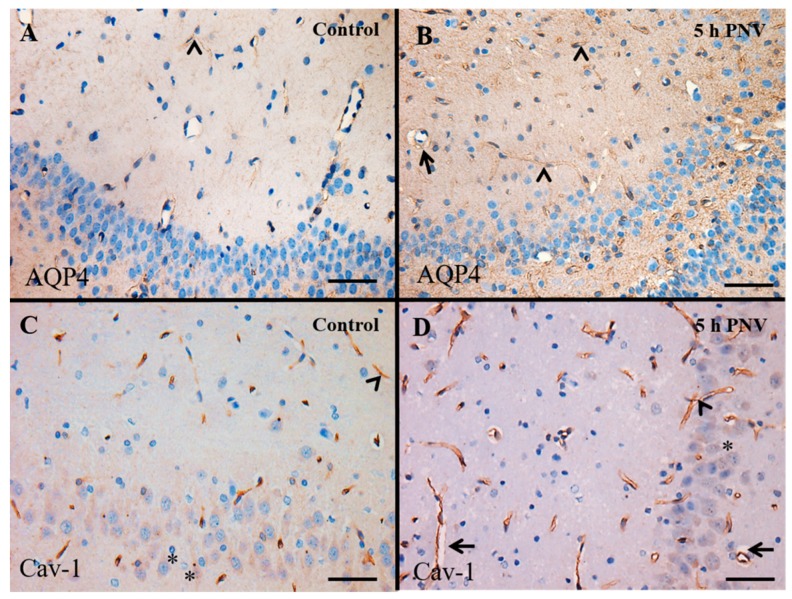
Illustrative immunohistochemical localization of aquaporin (AQP4) and caveolin-1 (Cav-1) in an adult animal administered saline (**A**,**C**) or *Phoneutria nigriventer* venom (PNV) (**B**,**D**) in CA3. (**A**,**C**) The AQP4 and Cav-1 physiologic expression in controls; and (**B**,**D**) PNV-induced up-regulated AQP4 and Cav-1, respectively. Arrowhead: vessel labeling. Arrow: perivascular edema formation in hippocampus capillaries of PNV-administered animals. Asterisk: neuronal labeling. Scale Bar: 100 µm for (**A**,**B**) and 50 µm for (**C**,**D**).

AQP4 immunoreactivity was weak in the hippocampi of controls (Figure 2A), suggesting that the brain water transport is at the physiological level. In PNV-treated samples, the water metabolism changed depending on the time-course post-envenoming. Figure 2B is illustrative of AQP4 in an adult animal 5 h post administration of PNV where the protein was upregulated; in this case, the expression was found mainly in thin astrocytes processes spread all over hippocampal regions and especially around the capillaries wall. Figure 2C illustrates the Cav-1 immunoreactivity in the endothelium of hippocampal capillaries in control animals. Figure 2D shows Cav-1 up-regulation in a PNV-treated adult animal both by stronger reactivity as well as by increased number of labeled capillaries and perikarya of pyramidal neurons. In addition, the rats administered PNV showed perivascular edema (Figure 2B,D), likely as a result of swollen astrocytic perivascular end-feet, while no such event is found in saline-administered animals (Figure 2C). The findings suggest alteration of water metabolism and BBB permeation disturbance.

### 2.3. AQP4

**Figure 3:**
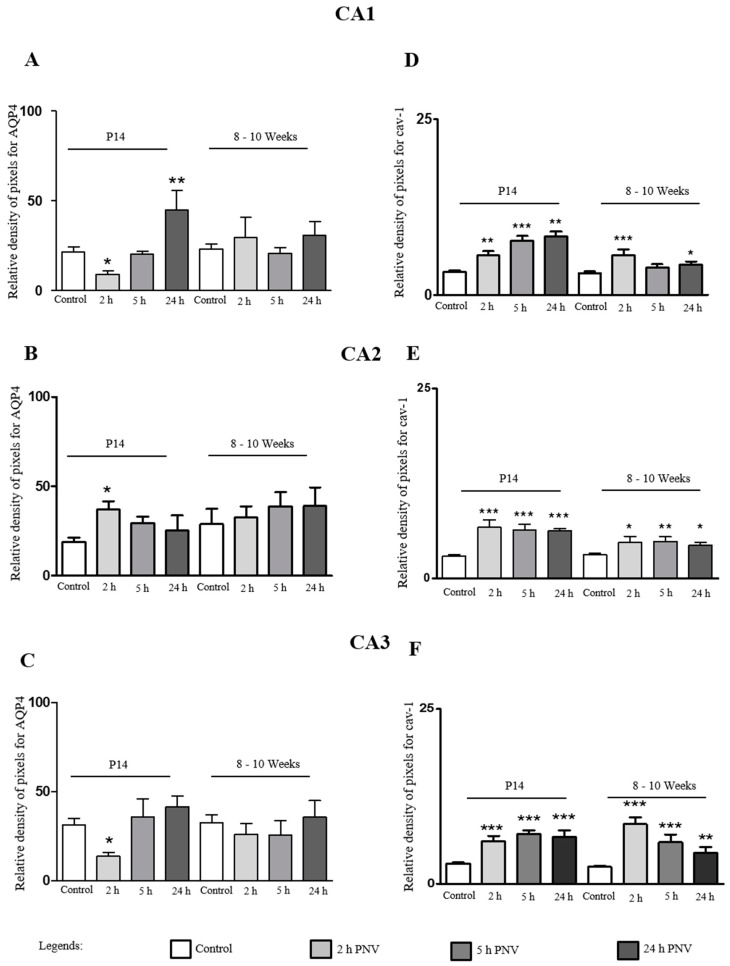
Percentage of pixels density of aquaporin-4 (APQ4) and caveolin-1 (Cav-1) labeled cells after PNV or 0.9% saline injection; immunohistochemistry of AQP4 and Cav-1 was computer-treated in order to segment by labeling color the immunoreactive sites of each protein through the use of GIMP 2.6.4 software. (**A**–**C**) Graphs of neonates and adults for AQP4 protein expression after envenoming in CA1, CA2 and CA3 hippocampal subfields; (**D**–**F**) Graphs of neonates and adult rats for Cav-1 expression after envenoming in CA1, CA2 and CA3. * *p* < 0.05, ** *p* < 0.01 and *** *p* < 0.001 compared to the saline control (Student’s *t*-test of data expressed as mean ± SEM); *n* = 10 images per subfield, per treatment, per time-point.

CA1: In CA1 region of PNV-administered neonate rats, AQP4 expression was episodically down-regulated at 2 h after envenomation (140%, *p* < 0.05), a period marked by severe toxic manifestation of animals; then returned to basal levels at 5 h, when animals started showing signs of recuperation; and increased again at 24 h (108%, *p* < 0.01), a period at which animals show no visible sign of intoxication. In adult animals, no significant alterations were detected in all time points assessed, most likely due to individual variability (large SEM bars) (Figure 3A).

CA2: The water channel protein was up-regulated in all time-points both in P14 and 8–10 week-old rats, with difference that the up-regulation was just significant at 2 h for P14 (52%, *p* < 0.05) (Figure 3B). Figure 3 shows the time-course quantification of the density of pixels after selection by color segmentation of proteins labeling. The data were expressed as percentage of AQP4-labeled astrocytic processes, and Cav-1-labeled endothelia in CA1, CA2 and CA3 sub-fields of hippocampi in saline- (control) and PNV-treated rats.

CA3: In CA3 region, a 58% down-regulation of AQP4 at 2 h (*p* < 0.05) followed by return to baseline (5 h) and a non-significant increase at 24 h. In contrast, in adults, none of the time-points post envenoming showed alterations in comparison to control group (Figure 3C).

The measurement of pixels’ density shows that basal AQP4 immunoreactivity in P14 rats was higher in the order CA3 > CA1 > CA2; PNV exposure altered the immunoreactivity grade seen in controls. In PNV-treated rats, CA1 was the subfield where downregulation (2 h) and upregulation (24 h) of the protein were highest. At 2 h time after envenomation, when toxic condition of animals was worst, the expression of AQP4 in P14 was significantly changed in all subfields CA1, CA2 and CA3. At 24 h after PNV, AQP4 expression had returned to control level or was upregulated.

### 2.4. Cav-1

Expressional changes were evident in all three hippocampal sub-fields of neonate and adult rats in response to PNV *intra peritoneal* exposure. Nevertheless, the proportion of up-regulation was lower in adults than in neonate P14 rats. Significant increases in Cav-1 expression in P14 vs. adult rats per time-course/region were: at 2 h (124% vs. 81% for CA1; 128% vs. 57% for CA2; 111% vs. 253% for CA3, respectively), 5 h (209% vs. 25% for CA1; 118% vs. 60% for CA2; 146% vs. 146% for CA3, respectively) and 24 h (232% vs. 39% for CA1; 114% vs. 45% for CA2 and 140% vs. 84% for CA3, respectively) post PNV-injection.

The up-regulation of the transmembrane protein Cav-1 in the endothelium plasma membrane, in response to PNV is indicative of increased formation of caveolae and endocytosis at the BBB, as well as is also suggestive of increased cell trafficking and signal transduction mediated by caveolae.

## 3. Discussion

The main findings from this study are that the sub-lethal dose of the venom of *P. nigriventer* spider injected in rats produces neuro-excitotoxic manifestations to the animals (P14 and 8–10 week-old) which paralleled with disturbances to hippocampal AQP4 and Cav-1, two transporter proteins and specific markers of astrocytes and endothelial cells, respectively; Second, the intensity and duration of the toxic manifestations indicate P14 neonate rats more vulnerable to PNV than adults; Third, the intensity of the toxic manifestations to P14 decreases temporally while inversely the expression of AQP4 and Cav-1 increases peaking at 24 h when clinically animals appearance do not differ from saline controls; Forth, histologically the capillaries of P14 and 8–10 week-old rats treated with PNV show perivascular edema, while controls did not; Fifth, immunohistochemistry reveals that PNV affects CAs sub-fields of hippocampus at different degrees throughout time-points; Sixth, regional immunohistochemistry quantification reveals that in P14 rats Cav-1 peaks at 24 h when toxic manifestations are vanished, whereas, in 8–10 week-old rats, Cav-1 peaks at 2 h when animals present intense toxic signs and progressively attenuates such increases until 24 h, remaining though significantly above baseline; Lastly, this study demonstrates that both the alterations provoked by PNV to astroglial AQP4 and endothelial Cav-1 in the hippocampus are involved in the mechanisms of BBB breakdown induced by PNV, first reported by Le Sueur and co-workers [8,9]. The unique characteristics of endothelium and astrocytic end-feet processes as well as the intimacy of both glio-vascular cell components at the blood–brain interface are the determinants for microenvironment homeostasis and steady neuronal function. Toxicological conditions of animals and disturbance of AQP4 and Cav-1 proteins in animals, mainly P14, treated with sub-lethal dose of PNV indicate that homeostasis was no longer working properly.

AQP4 and Cav-1 upregulation has been associated to BBB impairment and edema formation and/or resolution, neuron excitability, endocytosis and neuroinflammation [20,26,28,29,30,31].

The venom of *P. nigriventer* causes BBB breakdown, swelling of perivascular astrocytes and extravasation of fluid into the brain extracellular spaces indicating edema formation and BBB impairment in cerebellum and hippocampus [8,10,32]. PNV also activates neurons, and induces expression of GFAP, S100 and pro-inflammatory cytokines in astrocytes of the cerebellum and hippocampus with evidences of nitric oxide (NO) derived from neuronal and endothelial nitric oxide synthase (nNOS, eNOS, respectively) mediation [33,34,35].

The present study shows through different assays that two transporter proteins, AQP4 and Cav-1, undergo changes in the hippocampus of rats after PNV envenomation. Moreover, hippocampal microvasculature of PNV-treated rats shows perivascular edema, likely a reflex of swollen perivascular astrocytic end-feet. The findings match with previous studies using same experimental design and animal model that have shown that PNV induces up-regulations of AQP4 [12] and Cav-1 [13] in the cerebellum. Thus, likewise in the cerebellum, this study reveals that both the metabolism of water and the endocytosis and trafficking via fluid-phase transcytosis is age- and region-relatedly altered. 

The AQP4 immunoreactivity was found in astrocytes processes throughout the hippocampal parenchyma and more markedly at the capillary wall surroundings. In addition, the Cav-1 immunoreactivity was found delineating finely the endothelium of the hippocampal capillaries. The findings conforms to the AQP4 expression, especially at the end-feet membrane specialized domains of perivascular astrocytes, and in close contact with blood vessels endothelium [16,36]. Cav-1 is a typical endothelial protein, representing 95% of the vesicles in endothelial membrane [37]. The isoform α of Cav-1 is ubiquitously a marker of the protein in the brain [25]. Cav-1 is the main structural protein of caveolae, a vesicle-shaped specialized lipid-raft domain of endothelium plasma membrane rich in cholesterol and glycosphingolipids [38], signaling Src kinase [39] and eNOS; the increased expression of Cav-1 has been associated with increased formation of caveolae along the endothelial plasma membrane, increased endocytosis and transcytosis and destabilization of BBB permeability, which collectively contribute to vasogenic edema [13,26,27,40]. In contrast, degradation of cholesterol by filipin [41] or Cav-1 deletion prevents caveolae-mediated endocytosis and transcytosis, neuronal death and prevention of vasogenic edema [42].

### 3.1. AQP4

Comparison of the dynamics of AQP4 expression and its mRNA content at the time frame selected indicates that P14 rats are more vulnerable to PNV than adults; we hypothesize that regulatory mechanisms relative to water metabolism have yet not completely settled down in astrocytes at this age. Indeed, functional properties of these glial cells can change during development and in response to neuropathological conditions [43]. In astrocytes of the cerebellum and Muller astrocytes of rats’ retina, AQP4 was detectable at the second week of postnatal life, and gains progressive increases peaking at P56 [44] and P60 [45] of postnatal life. Similar developmental regulation was found in the expression of AQP4 in the hippocampus of mice, which gradually increases from P9 through P21 and P42 days of postnatal life [46].

Apart from that, there is increasing evidence that astrocytes from different compartments have distinct pharmacological and immunocytochemical properties [43]. Studies have shown that in the mouse hippocampus, the highest expression of AQP4 was detected in astrocytes of CA1, and that AQP4 and Kir4.1 were co-expressed ubiquitously in nearly all CA1 astrocytes [46]. The authors identified CA1subfield as potential site of astrocytic K^+^ and H_2_O regulation, a critical issue in various brain diseases.

Such finding correlates with our immunohistochemistry quantification of AQP4 reactivity in P14 hippocampal astrocytes of rats injected with PNV. Herein, the CA1 was the subfield with major downregulation of AQP4 in response to PNV (140% in CA1 vs. 58% in CA3 at 2 h), and also the subfield with major upregulation (108%/24 h in CA1 vs. 52%/2 h in CA2). Astrocytes are key protagonists to maintain brain homeostasis, which involves the reuptake of both extracellular K^+^ and excitatory amino acids and Ca^2+^ balance after neuronal activity. PNV contains neurotoxins that activate or delay inactivation of Na^+^ and block Ca^2+^ and K^+^ channels and interfere in glutamate handling (see [6] for review). Further studies are necessary to determine the potential role of each subfield in the regulation of water metabolism. However, based on report by Hsu and co-workers [46] and on our findings we do not discard the possibility of CA1 as potential site of K^+^ buffering and water regulation in the hippocampus. Another interesting issue is to investigate whether the blockade of K^+^ channels by PNV has also cell and sub-field specificity, which could be related to differential changes in AQP4 expression. The presence of perivascular edema is indication of BBB breakdown and substantiates our suggestion that changes in expression of both AQP4 and protein transcripts reflects disturbance in water balance in the hippocampus. The present results indicate PNV as useful tool in the study of diverse diseases related with brain edema and excitability.

### 3.2. Cav-1

The different degrees of up-regulation of Cav-1 at the time frame examined implies a changeable caveolae dynamics wherein the assembly/disassembly cycles are transformed in mechanical tension over the endothelial membrane, induces shear stress and consequent interference in hemodynamics [47,48]. The Cav-1 protein from caveolae, apart from its role in transporter mechanisms, is more recently implicated in sensing mechanical forces underwent by endothelial membrane, formation and stabilization of synapses, intercellular communication and signal transduction, always involving calcium signals [24,48,49]. In this study, the Cav-1 immunoreactivity of pyramidal neurons of CA hippocampal subfields is documental for the role of the protein also in neuronal cells function; PNV seemed to enhance the protein labeling (compare the intensity of Cav-1 labeling of pyramidal neurons pictured in Figure 3C (control) and Figure 3D (PNV-treated)). In hippocampal neurons, there is indication that induction of Cav-1 is triggered through glutamate exposure, apparently through kainate and AMPA-type receptors [50], receptors that are involved in hypernociception caused by PNV [6,7,51,52].

In the endothelium, Cav-1 and eNOS share same caveolae’s membrane micro-domain; Cav-1 negatively regulates eNOS [53]. In the present study, the rise in the expression of Cav-1 (WB and IHC data) promoted by PNV suggests eNOS inhibition and poor availability of oxide nitric (NO) and thus endothelium dysfunction. Recent study of our group has shown that PNV proper reduces availability of NO by leading to eNOS uncoupling [35]; at early periods following envenomation when toxic manifestation is severe, there is monomerization of eNOS and incapacity of the enzyme to produce NO, leading to superoxide production and endothelial/vascular BBB dysfunction [54].

Taken together, these data indicate that the two transporter proteins, AQP4 and Cav-1, are targets of PNV thus revealing their participation in the mechanism involved in the BBB breakdown in the hippocampus’s capillaries. Considering the preponderance of PNV effects in P14 compared with 8–10 week-old rats we suggest the existence of differences in regulatory mechanisms related to AQP4 and Cav-1/caveolae system. Considering the astrocyte-endothelial intimate physical and functional interactions and permeability of the BBB [55], we hypothesize that age-related modulations might be linked to changes in functional properties of astrocytes during post-natal development [43] with implications to the endothelium Cav-1/caveolae system. The age-related differences here seen could have role in the higher severity of accidents victimizing children than adults. Neonates are known to be more susceptible to pathological conditions of the brain than adults [56].

## 4. Materials and Methods

### 4.1. Venom and Animals

The lyophilized venom of *P. nigriventer* spider was kindly donated by Evanguedes Kalapothakis from Department of General Biology, Institute of Biological Sciences, Federal University of Minas Gerais (UFMG), Belo Horizonte, MG, Brazil and maintained in −20 °C until the moment used.

Male Wistar rats (*Rattus norvegicus*) of two ages, 14 days (P14, neonate group) and 8–10 weeks (8–10 weeks, adult group), were obtained from the Animal House Center Service (CEMIB) at Unicamp, Campinas, SP, Brazil. The animals were kept in room temperature with food and water ad libitum and 12/12 h cycles of day/night.

The study was approved by the institution’s Committee for Ethics in Animal Use (CEUA-UNICAMP, protocols 2405-1 and 2411-1) which follows the Brazilian Society for Laboratory Animal Science (SBCAL) guidelines.

### 4.2. Envenoming Procedure

The experiments consist of applying an intraperitoneal (i.p.) and sublethal injection of PNV (1.7 mg/kg) diluted in sterile saline (0.5 mg/µL) according to the weight of the animal. The 1.7 mg/kg dose was the one that better reproduced the sub-lethal signs of envenoming [8]. Three times points related to grade of toxic manifestation after venom exposure were chosen: 2 h (intense envenoming signs); 5 h (signs of recovery from toxic manifestation); and 24 h (absence of clinical neurotoxic manifestation). There were in total *n* = 5 animals per hour/technique (total *n* = 45 per age). Pared controls received the same volume of the vehicle (0.9% saline) (*n* = 45 per age/technique/hour). The sacrifice procedures were described below at each techniques section. 

### 4.3. IHC

The animals were euthanized with anesthetics overdose (3:1 mixture of ketamine chloride (Dopalen^®^, 100 mg/kg body weight) and xylazine chloride (Anazedan^®^, 10 mg/kg body weight, both from Fortvale, Valinhos, SP, Brazil) and perfused through the left ventricle with 150 mL of physiological saline followed by 250 mL of 4% paraformaldehyde in 0.1 M phosphate-buffered saline (PBS), pH 7.4. Then, the hindbrain was immediately removed, post-fixed in the same fixative overnight, dehydrated in a graded ethanol series, cleared in xylene and embedded in Paraplast^®^ (Sigma-Aldrich, St. Louis, MO, USA). The immunohistochemistry was performed in 5 µm thick sections [11]. Briefly, after dewaxed, dehydration and water rinsing, the endogenous peroxidase was blocked and antigen retrieval was performed. Then, the sections were incubated overnight with primaries antibodies for anti-rabbit AQP4 (1:100, Sigma-Aldrich) and Cav-1 isoform α (1:200, SC-894, Santa Cruz, CA, USA). The HRP enzyme (Kit Envision, Dako, Agilent Technologies Company, Glostrup, Denmark) was used as secondary antibody and DAB (Dako Cytomation, CA, USA) as a chromogenic solution. The slides were counterstained with Harris Hematoxylin and mounted in Canada balsam. Images were captured with a photomicroscope Olympus BX51 (Olympus Corporation, Tokyo, Japan). One percent BSA was used as substitute for primaries antibodies as negative control of the reaction.

#### IHC Analysis

**Figure 4:**
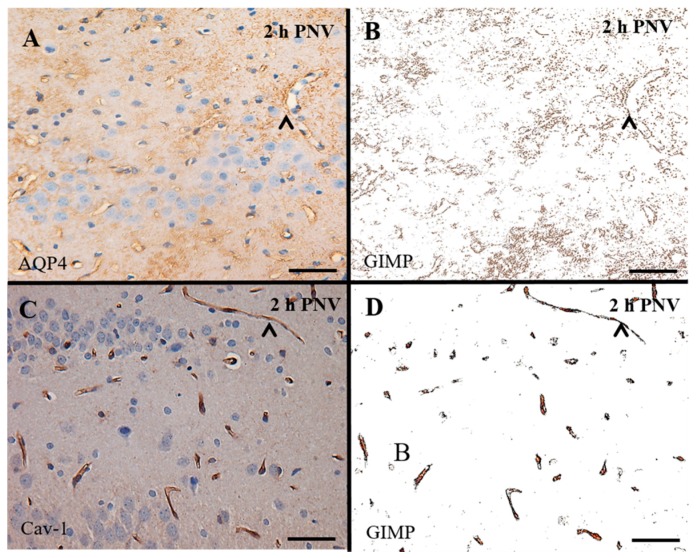
Illustrative image analysis using 2.6.4 GIMP software: (**A**,**B**) CA3 region of neonate rat 2 h post-PNV labeled with aquaporin-4 (AQP4) and caveolin-1 (Cav-1) antibody (brown) and counterstained with Harris Hematoxylin; and (**C**,**D**) segmentation by color of the primary antibody reactivity analyzed by 2.6.4 GIMP software for further quantification of density of pixels. (**A**,**B**) The widespread labeling refers to labeling of astrocytic distal processes; arrowhead shows endothelial labeling; and (**C**,**D**) labeling of Cav-1 in capillaries endothelium. Scale bars: 50 µm.

In order to quantify alterations in AQP4 and Cav-1 expression, immunolabeling of proteins was measured using the GIMP 2.6.4 (GNU Image Manipulation Program software, CNET Networks, Inc., Washington, PA, USA) that converts the digitized images to grayscale images (black and white) after color selection [57]. Digitized images were taken using a 40× objective using the same aperture and illumination parameters for all. Two random fields of each region of hippocampus (CA1, CA2, CA3) for each animal (*n* = 5/per time/age), totaling 10 images per region per treatment (PNV and 0.9% saline), were selected. Figure 4 illustrates immunoreactivity of AQP4 and Cav-1 in hippocampal CA3 sub-field and corresponding image after color segmentation in order to transform it in density of pixels.

### 4.4. qPCR

After the animals were submitted to CO_2_ chamber, heads were cut off and the brains rapidly removed and dissected. The hippocampi were homogenate using Trizol reagent (Life Technologies, Gaithersburg, MD, USA), RNA of hippocampus was extracted according to the manufacturer’s recommendations. Standard reverse-transcription PCR was performed using total RNA as described previously [58]. Intron-skipping primers (TaqMan^®^ Universal Master Mix) for AQP4 (catalogue number: Rn00563196_m1) and Cav-1 (catalogue number: Rn00755834_m1) were obtained from Applied Biosystems (Carlsbad, CA, USA). Glyceraldehyde-3-phosphate dehydrogenase (GAPDH) primer (Applied Biosystems) was used as control. qPCR analysis of gene expression was performed in an ABI Prism 7500 sequence detection system (Applied Biosystems). The optimal concentrations of cDNA and primers, as well as the maximum efficiency of amplification, were obtained through five-point, two-fold dilution curve analysis for each gene. Each PCR contained 25 ng of reverse-transcribed RNA, 200 nM of each specific primer, SYBR SAFE PCR master mix, RNase free and water to complete a final volume of 20 µL. Real-time data were analyzed using the Sequence Detector System 1.7 (Applied Biosystems).

### 4.5. Western Blotting (WB)

The animals were submitted to carbon dioxide (CO_2_) chamber, heads were cut off in a guillotine, the hindbrain rapidly removed and stored in liquid nitrogen. The hippocampus were dissected and homogenized with anti-protease cocktail (10 mM EDTA, 2 mM PMSF, 100 mM NaF, 10 mM sodium pyrophosphate, 10 mM NaCO4, 10 g of aprotinin/mL and 100 mM Tris, pH 7.4), the contents spun 3000 rpm for 10 min and the supernatant collected and stored at −80 °C. The protein concentration was determined with a kit from Bradford protein assay (Bio-Rad Laboratories Inc., Hercules, CA, USA). The SDS/PAGE were done with 12% polyacrylamide gel for AQP4 (34 kDa) and Cav-1 (23 kDa). After electrotransfer to a nitrocellulose membrane, the membranes were incubated with basal solution (0.1% Tris-buffered saline with 0.05% Tween 20, pH 7.4) plus 5% dry milk for two hours at room temperature. Then, the membranes were placed with the primaries antibodies presented in IHC technique for 5 to 18 h (1:2000 for AQP4 and 1:1500 for Cav-1) followed anti-rabbit secondary antibody (1:4000, Santa Cruz Biotechnology, Santa Cruz, CA, USA) for two hours. To visualize the proteins bands a chemiluminescence kit (Super Signal West Pico Chemiluminescent Substrate; Pierce Biotechnology, Rockford, IL, USA) and an X-ray film (Sigma-Aldrich) was used. The NIH Image J program 1.41 (developed by Wayne Rasband, NIH, Bethesda, MD, USA) software was used to performed the densitometry analyses. β-actin (Sigma-Aldrich) immunoblot was done as an internal control. The results were confirmed in three sets of experiments.

### 4.6. Statistical Analysis

All data were expressed as the mean ± standard error of the mean (SEM) and were analyzed using the Graphpad Prism software package (San Diego, CA, USA). The statistical significance among control and PNV-treated groups was determined by unpaired Student *t*-test with value of *p* < 0.05 indicating statistical significance.

## 5. Conclusions

Taken together, the data indicate that the two transporter proteins, AQP4 and Cav-1, are targets of PNV thus revealing their participation in the mechanism involved in the BBB breakdown in the hippocampus’s capillaries. Considering the preponderance of PNV effects in P14 compared with 8–10-week-old rats, we suggest the existence of differences in regulatory mechanisms related to AQP4 and Cav-1/caveolae system. In addition, considering the astrocyte-endothelial intimate physical and functional interactions and BBB permeability [55], we hypothesize that age-related modulations here found might be linked to changes in functional properties of astrocytes during post-natal development [43], with implications to the endothelium Cav-1/caveolae system. The age-related differences could have role in the higher severity of accidents victimizing children than adults. Further studies to identify CA1 subfield as a main water regulatory center in the hippocampus in response to PNV are needed.
